# Jian-Pi-Yi-Shen Formula ameliorates chronic kidney disease: involvement of mitochondrial quality control network

**DOI:** 10.1186/s12906-018-2395-2

**Published:** 2018-12-20

**Authors:** Xinhui Liu, Jianping Chen, Xiaoyan Liu, Dongtao Wang, Ping Zheng, Airong Qi, Tiegang Yi, Shunmin Li

**Affiliations:** 1 Department of Nephrology, Shenzhen Traditional Chinese Medicine Hospital, Guangzhou University of Chinese Medicine, Shenzhen, Guangdong China; 2 Shenzhen Key Laboratory of Hospital Chinese Medicine Preparation, Shenzhen Traditional Chinese Medicine Hospital, Guangzhou University of Chinese Medicine, Shenzhen, Guangdong China

**Keywords:** Traditional Chinese medicine, Chronic kidney disease, Fibrosis, Jian-Pi-Yi-Shen formula, Mitochondrial quality control network

## Abstract

**Background:**

Jian-Pi-Yi-Shen Formula (JPYSF), a Chinese herbal decoction with the efficacies of ‘fortify the spleen and tonify the kidney’ and ‘activate blood and resolve stasis’, is effective for the treatment of chronic kidney disease in clinic. However, the underlying mechanism remains unclear. The aim of this study was to investigate the therapeutic effects and possible mechanisms of JPYSF on retarding chronic kidney disease progression in 5/6 nephrectomized (5/6 Nx) rats.

**Methods:**

Perindopril (4 mg/kg/d) and JPYSF (2.72 g/kg/d) were administrated by gavage to 5/6 Nx rats daily for 6 weeks. The therapeutic effects of JPYSF were evaluated by renal function, pathological injury, and fibrosis. The protein levels associated with mitochondrial quality control network were measured by Western blot and immunofluorescence analysis.

**Results:**

5/6 Nx rats showed obvious decline in renal function as evidenced by increased serum creatinine, blood urea nitrogen, and urinary protein excretion, and significant injury in kidney structure as evidenced by glomerular hypertrophy, tubular atrophy, and interstitial fibrosis. Administration of JPYSF for 6 weeks could improve renal function and ameliorate kidney structure injury in 5/6 Nx rats. Furthermore, the remnant kidneys of 5/6 Nx rats showed unbalanced mitochondrial quality control network manifested as decreased mitochondrial biogenesis, fusion, and mitophagy, and increased mitochondrial fission. Treatment of JPYSF could restore aforesaid aspects of mitochondrial quality control network.

**Conclusions:**

These results indicate that JPYSF can notably ameliorate 5/6 Nx-induced chronic kidney disease, which may be related with modulation of mitochondrial quality control network.

## Background

Chronic kidney disease (CKD) is a common chronic disease with an estimated global prevalence of approximately 8–16% [1]. Despite this, there are relatively few therapies in developing for the treatment of CKD. For patients with CKD, the role of the renin-angiotensin system modulation only exerts partial salutary effects and can not necessarily prevent the progression to end-stage renal disease and the need for renal replacement therapy [2, 3]. The limit option for CKD treatment has prompted patients to seek out some alternative strategies such as traditional Chinese medicines (TCM) [4–8]. The prevalence of CKD in China is 10.8% [9], and TCM is widely used for CKD treatment in China [10–13]. However, the question of TCM for CKD patients remains a matter of debate. A recent study provided solid evidence of the beneficial effects of prescribed TCM on CKD patients in Taiwan [14] supporting that TCM can be an attractive area for the development of therapeutic drugs on CKD.

Jian-Pi-Yi-Shen Formula (JPYSF), a Chinese herbal decoction, is composed of eight herbs, that is Astragali Radix, Atractylodis Macrocephalae Rhizoma, Dioscoreae Rhizoma, Cistanches Herba, Amomi Fructus Rotundus, Salviae Miltiorrhizae Radix et Rhizoma, Rhei Radix et Rhizoma, and Glycyrrhizae Radix et Rhizoma Praeparata cum Melle. According to TCM theory, JPYSF possesses the efficacies of ‘fortify the spleen and tonify the kidney’ and ‘activate blood and resolve stasis’ [15]. JPYSF was combined and modified from two traditional herbal decoctions namely Da-Huang-Gan-Cao-Tang (DHGCT) and Yu-Ping-Feng-San (YPFS). DHGCT was recorded in Jin Gui Yao Lue by Zhongjing Zhang (150 B.C. to A.D. 219), which is considered to remove static blood or excessive fluid through the bowels. YPFS was described in Dan Xi Xin Fa by Danxi Zhu in Yuan Dynasty (A.D. 1279–1368), which is being used to replenish “Qi”. To induce purgation by DHGCT and to replenish “Qi” by YPFS could be applied for the treatment of CKD-associated urine toxins retention and low immune response. For over 20 years, JPYSF has been clinically prescribed as basic formula for the treatment of patients with CKD. Results of our previous clinical study suggested that JPYSF significantly improved kidney function of CKD especially mild-to-moderate CKD patients, as evidenced by reducing serum creatinine (Scr) and blood urea nitrogen (BUN) levels [16, 17]. However, the underlying action mechanism of JPYSF remains unidentified and needs to be investigated.

The kidney is a highly aerobic organ and is rich in mitochondria. Therefore, kidneys are exquisitely dependent upon, and susceptible to, being damaged by mitochondria [18]. Studies have shown significantly increased reactive oxygen species production and abnormal respiratory chain complex expression in peripheral blood mononuclear cells of CKD patients, thereby demonstrating the closely association between mitochondrial dysfunction and CKD [19, 20]. Recent studies have also demonstrated that mitochondria participate in CKD progression and mitochondrial dysfunction led to increased proteinuria [21, 22], uremic toxin retention [23, 24], NLRP3 inflammasome activation [25] and transforming growth factor-β expression [26]. Healthy mitochondria are essential for kidney and they are maintained by a mitochondrial quality control network including mitochondrial biogenesis, mitochondrial fission and fusion and mitochondrial autophagy (mitophagy) [27]. However, the alteration of mitochondrial quality control network in CKD is still unclear. In the present study, we hypothesized that abnormal mitochondrial quality control network occurs in CKD development, and JPYSF treatment could restore mitochondrial quality control network in rat with 5/6 nephrectomy (5/6 Nx). Perindopril is a commonly used angiotensin converting enzyme inhibitor in the treatment of CKD. Therefore, we included a positive control group treated by perindopril to evaluate the effect of JPYSF on improving kidney function and structure in 5/6 Nx rats. And we further investigated the role of JPYSF in modulating mitochondrial quality control network.

## Methods

### Chemicals and antibodies

Sodium danshensu (1), salvianolic acid B (2), echinacoside (3), liquiritin (4), acteoside (5), calycosin 7-O-β-glucoside (6), astragaloside IV (7), formononetin (8), and hesperidin (internal standard, ISTD) were purchased from National Institutes for Food and Drug Control (Beijing, China). The purity of all marker chemicals were determined to be no less than 98% by normalization of peak areas, as revealed by HPLC-DAD. HPLC grade acetonitrile was purchased from Merck (Darmstadt, Germany), and ultrapure water was prepared using a Milli-Q purification system (Molsheim, France). Other reagents used here were of analytical grade. Perindopril was purchased from Sigma-Aldrich (St Louis, MO, USA). The primary antibodies included rabbit anti-peroxisome proliferator-activated receptor-γ coactivator-1α (PGC-1α), rabbit anti-PTEN-induced putative kinase 1 (PINK1) (Novus, Littleton, CO, USA), mouse anti-mitochondrial transcription factor A (TFAM), rabbit anti-nuclear respiratory factor 1 (NRF-1) (Santa Cruz Biotechnology, Santa Cruz, CA, USA), rabbit anti-dynamin-related protein 1 (Drp-1), rabbit anti-mitofusin 2 (Mfn-2), rabbit anti-heat shock protein-60 (HSP-60), rabbit anti-cytochrome c oxidase subunit IV (COX-IV), mouse anti-α-tubulin (Cell Signaling Technology, Beverly, MA, USA), mouse anti-optic atrophy 1 (OPA-1) (BD Biosciences, San Jose, CA, USA), rabbit anti-Parkin (phospho S65), rabbit anti-fibronectin, rabbit anti-type IV collagen, rabbit anti-lysosomal-associated membrane protein 1 (LAMP-1), mouse anti-cytochrome c oxidase subunit I (COX-І), mouse anti-nicotinamide adenine dinucleotide dehydrogenase (ubiquinone)-1β subcomplex 8 (NDUFβ8) (abcam, Cambridge, MA, USA), mouse anti-Parkin, mouse anti-α-smooth muscle actin (α-SMA) (Sigma-Aldrich, St Louis, MO, USA), rabbit anti-ATP synthase subunit beta (ATP5B) (Aviva Systems Biology, San Diego, CA, USA), and mouse anti-glyceraldehyde-3-phosphate dehydrogenase (GAPDH) (proteintech, Wuhan, China). Horseradish peroxidase (HRP)-conjugated anti-mouse IgG and HRP-conjugated anti-rabbit IgG were purchased from Life Technologies (Carlsbad, CA, USA).

### Plant materials and preparation of JPYSF water extract

The herbal composition and proportion of JPYSF was summarized in Table 1. Raw herbs were purchased from Shenzhen Huahui Pharmaceutical Co., Ltd. (Shenzhen, China). The plant materials were authenticated by Shangbin Zhang, the executive manager of Pharmaceutical Department, Shenzhen Traditional Chinese Medicine Hospital (STCMH) based on their morphological characteristics. The voucher specimens were kept at STCMH. Assurance of quality control for all the materials was validated according to the Chinese Pharmacopeia (China Pharmacopoeia Committee, 2015). Raw herbs were weighed and boiled twice in 8 times of ddH_2_O (*w*/*v*) for 1 h per time. The extraction liquid was centrifuged (13,000 rpm, 10 min), the supernatant was kept at 4 °C and filtered through a 0.22 μm filter (Millipore Ireland Ltd., Ireland) before HPLC analysis. For animal studies, the extract was dried by freeze dryer and stored at − 80 °C. Before the treatment, the freeze-dried powder was re-dissolved with ddH_2_O to get JPYSF extract.

**Table 1:** The herbal composition and proportion of JPYSF

Botanical name	Herbal name	Chinese name	Voucher number	Dosage
*Astragalus membranaceus* (Fisch.) Bge. var. *mongholicus* (Bge.) Hsiao	Astragali Radix	Huang-Qi	2010015Z	30 g
*Atractylodes macrocephala* Koidz.	Atractylodis Macrocephalae Rhizoma	Bai-Zhu	2010024ZZ	10 g
*Dioscorea opposita* Thunb.	Dioscoreae Rhizoma	Shan-Yao	2010037Z	30 g
*Cistanche deserticola* Y.C. Ma	Cistanches Herba	Rou-Cong-Rong	2040056Z	10 g
*Amomum kravanh* Pierre ex Gagnep.	Amomi Fructus Rotundus	Dou-Kou	202086Z	10 g
*Salvia miltiorrhiza* Bunge.	Salviae Miltiorrhizae Radix et Rhizoma	Dan-Shen	2010006Z	15 g
*Rheum palmatum* L.	Rhei Radix et Rhizoma	Da-Huang	2010040Z	10 g
*Glycyrrhiza uralensis* Fisch.	Glycyrrhizae Radix et Rhizoma Praeparata cum Melle	Zhi-Gan-Cao	2010008ZZ	6 g

### Chromatographic conditions and instrumentation

Validation HPLC method was performed on a Shimadzu (Kyoto, Japan) LC-20AT system, which was equipped with a degasser, a binary pump, an autosampler and a diode array detector. The herbal extract was separated on Agilent ZORBAX SB-C18 (250 mm × 4.6 mm, 5 μm) column. The mobile phase was composed of acetonitrile (A) and 10 mmol/L ammonium acetate (B) using the following gradient program: 0–1.2 min, 5% A; 1.2–2 min, 5–20% A; 2–4 min, 20–40% A; 4–8 min, 40% A; 8–10 min, 40–95% A; 10–17 min, 95% A; 17–20 min, 5% A; the flow rate was 0.8 ml/min; the injection volume was 5 μL. A Shimadzu mass spectrum (LC-2020) equipped with an ESI ion source was operated in positive and negative modes, and the selected ion monitoring was used. The drying gas temperature was 350 °C; drying gas flow: 1.5 L/min; nebulizer pressure: 35 psi; capillary voltage: 3500 V. Shimadzu Mass workstation software was used for data acquisition and processing.

### Animals and experimental treatment

All animal experiments were conducted with protocols approved by the Ethics Committee of Guangzhou University of Chinese Medicine and in accordance with National Institutes of Health Guideline for the care and use of laboratory animals (NIH Publications No. 80–23, revised 1996). Eight weeks old, male Spraque-Dawley (SD) rats were purchased from Guangdong Medical Laboratory Animal Center (Foshan, China) and maintained in a specific pathogen-free (SPF) animal facility under a 12 h light/12 h dark cycle, with free access to food and water. The 5/6 Nx operation was performed in rats under anesthesia with sodium pentobarbital (50 mg/kg body weight, intraperitoneal injection) by ablation of upper and lower thirds of the left kidney and then removal of the right kidney 2 weeks later. The sham operation consisting of laparotomy and manipulation of the renal pedicles but without destruction of renal tissue was performed. Twelve weeks after the second surgery, 53 rats remained alive including 10 rats with sham-operated and 43 rats with 5/6 Nx-operated. Thirty-seven 5/6 Nx rats with significant higher Scr levels were randomly assigned to 3 groups with 10 rats per group: 5/6 Nx (distilled water); 5/6 Nx + Perindopril (4 mg/kg/d); 5/6 Nx + JPYSF (2.72 g/kg/d). The same volume of distilled water was given to sham group (*n* = 10). The dosage of perindopril was determined by referring to previous studies [28, 29]. The dosage of JPYSF was derived from clinical CKD patients and our preliminary experiments. After 6 weeks of treatment, all rats were anesthetized (sodium pentobarbital, 50 mg/kg body weight, intraperitoneal injection), and blood samples were obtained by cardiac puncture. The rats were euthanized by cervical dislocation without regaining consciousness. Kidneys were removed and preserved for histological analysis, Western blotting, and immunofluorescence analysis.

### Biochemical analysis

Urinary albumin, urinary total protein, urinary N-acetyl-β-D-glucosaminidase (NAG), urinary creatinine, Scr, BUN, serum albumin, alanine transaminase (ALT), and aspartate transaminase (AST) were measured using a BS-180 automatic biochemistry analyzer (Mindray, Shenzhen, China) following the manufacturer’s instructions.

### Histological examination

Renal pathological injury was evaluated using periodic acid-Schiff (PAS) and Masson’s trichrome stains. For quantitative analysis, 40–50 glomerular tuft area and 40–50 proximal tubular lumen cross-sectional area in each rat and five rats in each group were measured using Nikon NIS-Elements BR software version 4.10.00 (Nikon, Japan) to evaluate glomerular hypertrophy and tubular atrophy.

### Western blotting

Equal amounts of kidney cortex lysates were loaded and electrophoresed through 7, 10%, or 15% SDS-polyacrylamide gels and were then transferred to nitrocellulose membranes or polyvinylidene difluoride membranes (Millipore, USA). Following blocking in 5% non-fat milk for 1 h at room temperature, the membranes were incubated with primary antibodies at 4 °C overnight. Then, the membranes were incubated in HRP-conjugated secondary antibodies for 1 h at room temperature. HRP activity was visualized using Clarity Western ECL Substrate and a ChemiDoc MP Imaging System (Bio-Rad Laboratories, USA). Image Lab software version 5.1 was used for densitometric analysis (Bio-Rad Laboratories, USA).

### Immunofluorescence analysis

The paraffin-embedded kidneys were treated by dewaxed, rehydrated, antigen retrieval, and blocking. Then, the sections were stained with primary antibodies at 4 °C overnight followed by appropriate secondary antibodies. Nuclei were counterstained with the fluorescent dye 4′,6-diamidino-2-phenylindole (DAPI). In all cases, antibody negative controls were used to ensure the truth of positive staining. All images were captured by fluorescence microscope (Nikon, Japan).

### Statistical analysis

Data are shown as mean ± SEM. Statistical significance among groups were tested by one-way ANOVA and post hoc analysis with the Least Significant Difference (LSD) test or the Games-Howell test. *P* < 0.05 was considered statistically significant. Data were analyzed using SPSS statistics software (version 16.0, SPSS Inc., Chicago, IL, USA).

## Results

### Preparation of standardized JPYSF extract

**Fig. 1:**
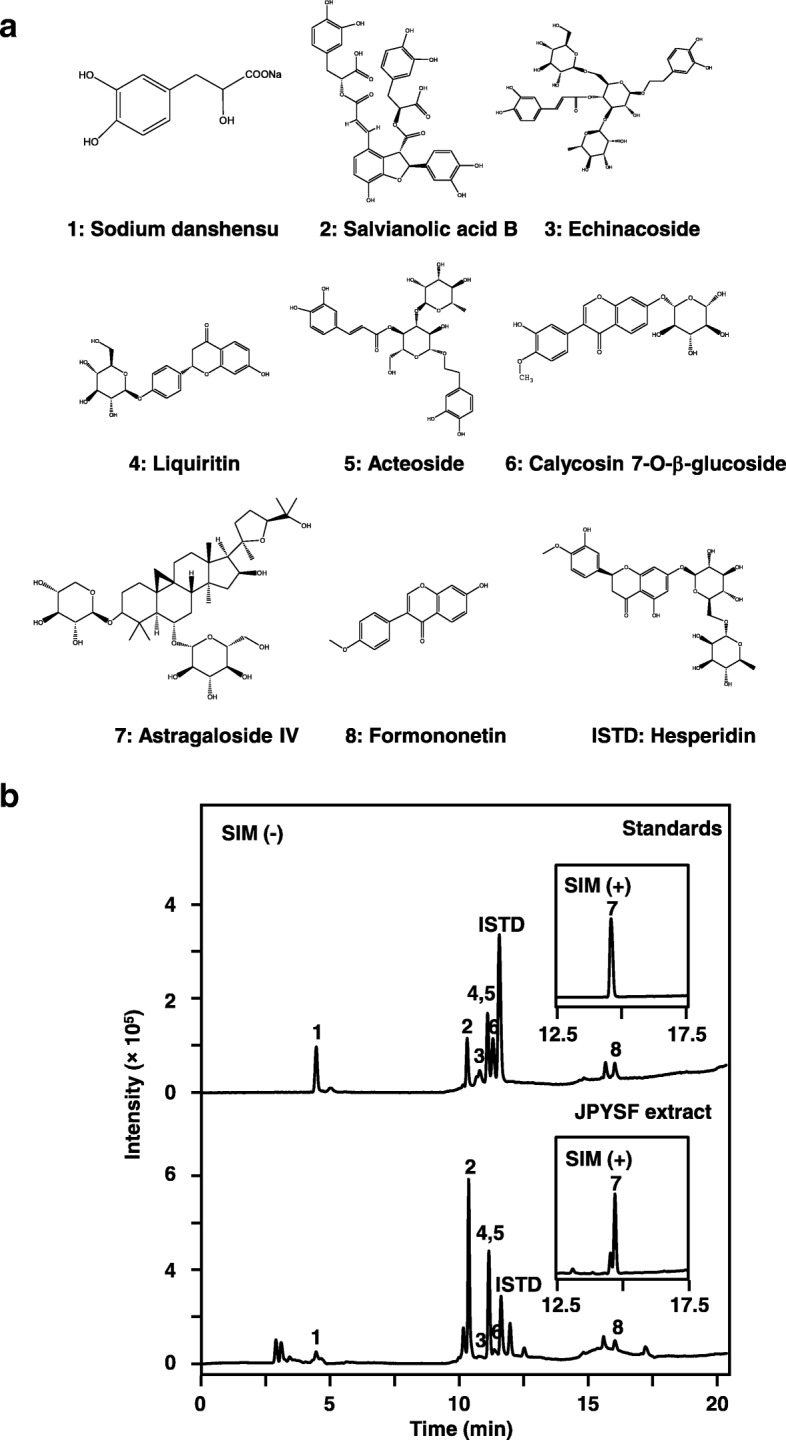
Typical LC-MS chromatogram of JPYSF. **a** Structures of chemical markers analyzed in JPYSF extract, including sodium danshensu (1), salvianolic acid B (2), echinacoside (3), liquiritin (4), acteoside (5), calycosin 7-O-β-glucoside (6), astragaloside IV (7), formononetin (8), and hesperidin (internal standard, ISTD). **b** The representative LC-MS chromatograms of mixed standards and JPYSF extract. The LC condition was described in the Methods. The denotations from 1 to 8 in the chromatogram correspond to the chemical markers as shown in (**a**). The identification of the chemical markers was determined by a MS detector in a negative mode except astragaloside IV (7) in a positive mode. Representative chromatograms were shown, *n* = 3

In order to chemically standardize JPYSF extract, we established an HPLC-MS method to reveal its HPLC profile and quantify the main components. Eight chemical markers were found in JPYSF extract (Fig. 1a). A typical HPLC-MS profile was developed for JPYSF extract (Fig. 1b), which served as an index for the identification of JPYSF. Moreover, eight chemical markers were quantified in the JPYSF extract defining the minimum requirement for 1 g of dried powder of standardized JPYSF, i.e., sodium danshensu (0.45 mg/g); salvianolic acid B (1.80 mg/g); echinacoside (0.50 mg/g); calycosin 7-O-β-glucoside (0.68 mg/g); acteoside (0.10 mg/g); liquiritin (0.60 mg/g); astragaloside IV (0.05 mg/g); formononetin (0.60 mg/g). The yield of the extraction was ~ 32.59 ± 1.1% (*w*/w, Mean ± SD, *n* = 3). The extract being used here reached the aforesaid requirements, which guaranteed the repeatability of biological results.

### Body weight and biochemical profiling

**Fig. 2:**
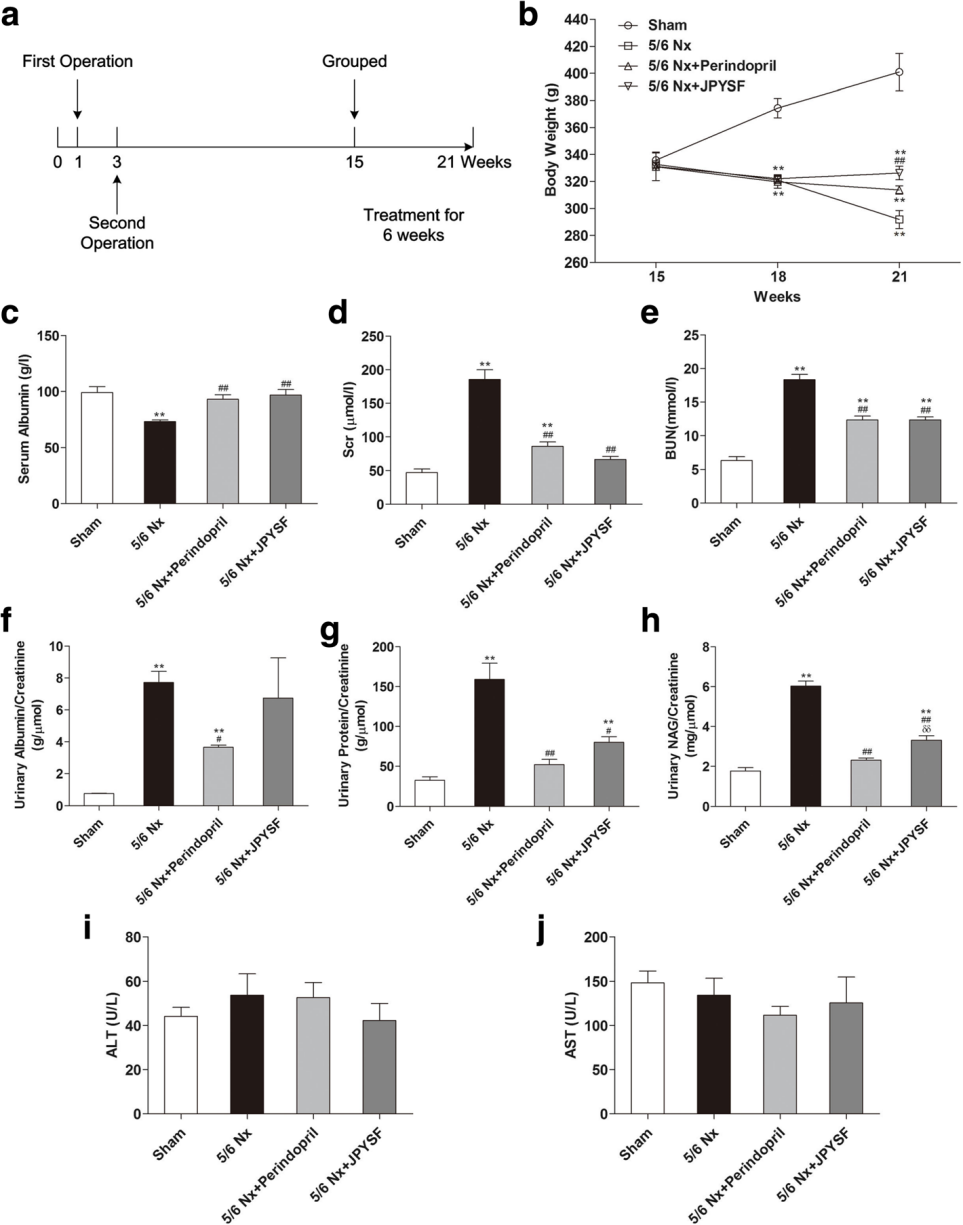
Body weight and biochemical indexes. **a** Timeline of study process. **b** Body weight changes during experiment. **c** Serum albumin levels. **d** Scr levels. **e** BUN levels. **f** Urinary albumin excretion adjusted by urinary creatinine. **g** Urinary total protein excretion adjusted by urinary creatinine. **h** Urinary NAG excretion adjusted by urinary creatinine. **i** Serum ALT levels. **j** Serum AST levels. Data are presented as the means ± SEM, *n* = 6 rats per group. (**P < 0.01 compared with the sham group; ^#^*P* < 0.05, ^##^*P* < 0.01 compared with the 5/6 Nx group; ^δδ^*P* < 0.01 compared with the 5/6 Nx + Perindopril group)

The timeline of present study was showed in Fig. 2a. Compared with sham group, rats in the 5/6 Nx groups showed substantial decline in body weight throughout the experiment. Administration of JPYSF could retard weight loss in 5/6 Nx rats at the final 6 weeks for treatment (*P* < 0.01) (Fig. 2b). Rats in the 5/6 Nx group showed lower serum albumin level, higher Scr and BUN levels, which could be restored by perindopril or JPYSF treatment (Fig. 2c-e). In urinary marker respect, the decrease of urinary albumin excretion was not as significant as perindopril treatment in 5/6 Nx + JPYSF group (Fig. 2b). However, both urinary total protein and NAG levels were significantly reduced following treatment of perindopril or JPYSF (Fig. 2g and h). The levels of serum ALT and AST were not significantly different among four groups (Fig. 2i and j).

### JPYSF ameliorated renal pathological injury in 5/6 Nx rats

**Fig. 3:**
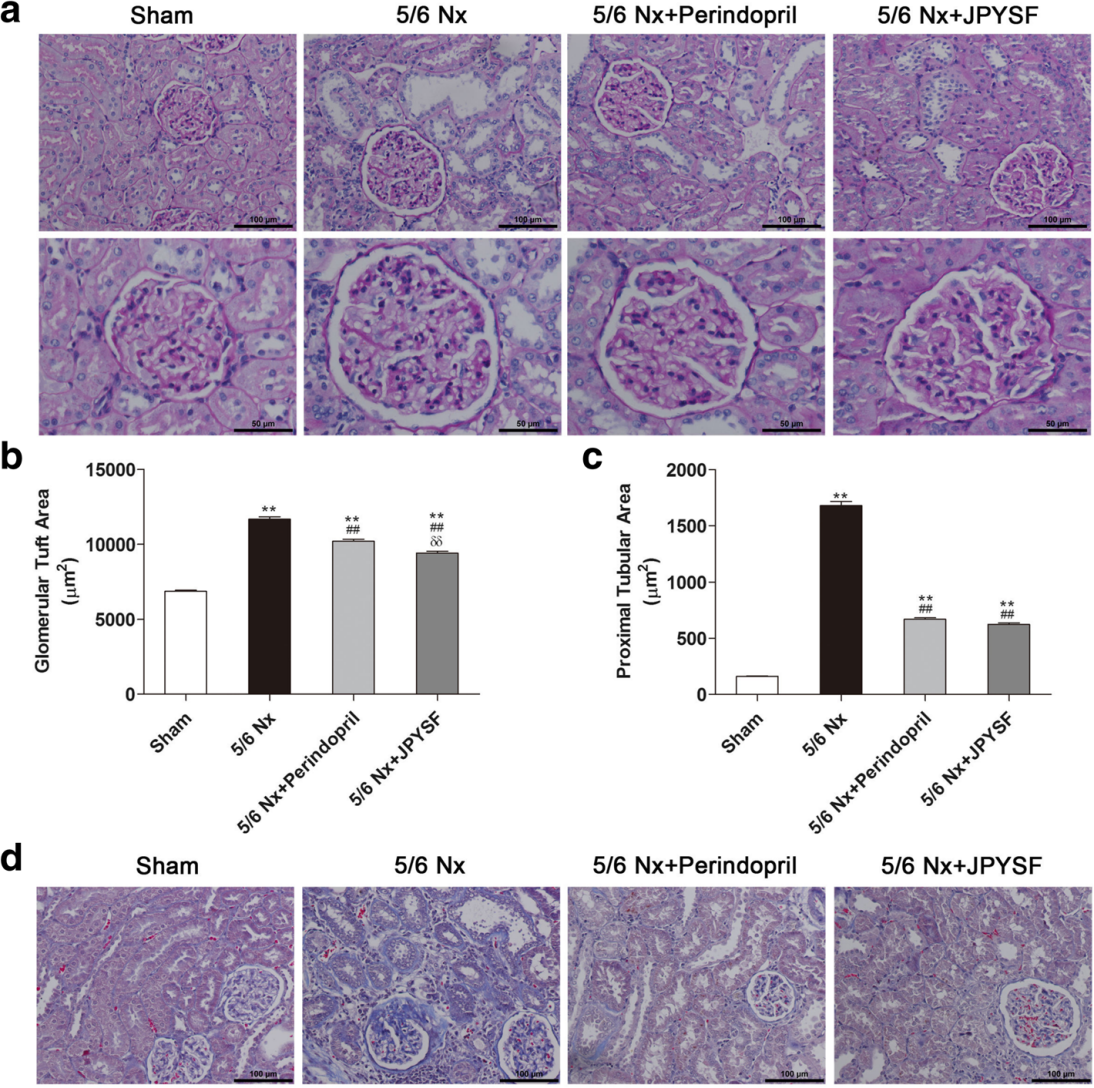
JPYSF ameliorates renal pathological injury in 5/6 Nx rats. **a** PAS staining shows significant improvement of glomerular hypertrophy and tubular atrophy in the 5/6 Nx + JPYSF group. All images are shown at identical magnification, the upper panel × 200, scale bar = 100 μm; the lower panel × 400, scale bar = 50 μm. Quantitative analyses of (**b**) glomerular tuft area and (**c**) proximal tubular lumen cross-sectional area in each group. **d** Masson staining indicates reduced fibrosis after JPYSF treatment. All images are shown at identical magnification, × 200, scale bar = 100 μm. For (**b**) and (**c**), 40–50 glomerular tuft area and 40–50 proximal tubular lumen cross-sectional area in each rat and five rats in each group were measured. Data are presented as the means ± SEM, *n* = 5 rats per group. (**P < 0.01 compared with the sham group; ^##^*P* < 0.01 compared with the 5/6 Nx group; ^δδ^*P* < 0.01 compared with the 5/6 Nx + Perindopril group)

PAS-stained sections revealed normal kidney structure in the sham group. In contrast, prominent glomerular hypertrophy and tubular atrophy were observed in the 5/6 Nx group, which were further proved by quantitative analyses. Administration of perindopril or JPYSF significantly ameliorated pathological injury occurred in 5/6 Nx rats (Fig. 3a-c). Apart from glomerular and tubular injury, rats in the 5/6 Nx group also showed obvious interstitial fibrosis observed in Masson-stained sections, which was improved by perindopril or JPYSF treatment (Fig. 3d).

### JPYSF down-regulated fibrosis-associated protein expression in 5/6 Nx rats

**Fig. 4:**
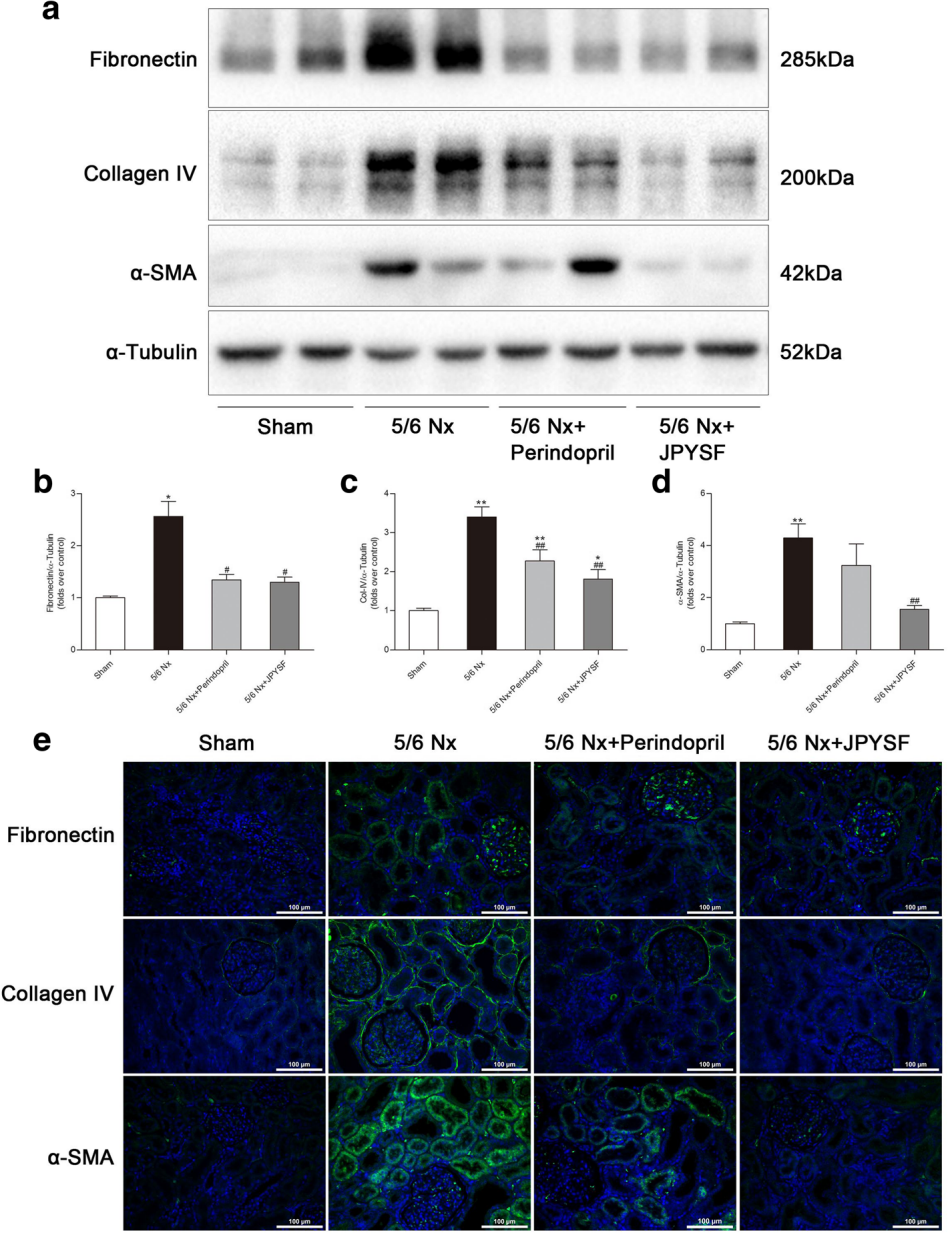
JPYSF retards renal fibrosis in 5/6 Nx rats. **a** Representative Western blot images indicate that fibronectin, collagen IV, and α-SMA protein expression were significantly increased in the 5/6 Nx group but were abolished by JPYSF treatment. **b**-**d** Densitometric analysis of fibronectin, collagen IV, and α-SMA protein expression normalized to α-tubulin content. **e** Representative immunofluorescence images of fibronectin, collagen IV, and α-SMA. Green corresponds to interest proteins, and blue corresponds to nuclear staining. All images are shown at identical magnification, × 200, scale bar = 100 μm. Data are presented as the means ± SEM, *n* = 6 rats per group. (*P < 0.05, **P < 0.01 compared with the sham group; ^#^*P* < 0.05, ^##^*P* < 0.01 compared with the 5/6 Nx group)

Western blotting revealed that the expression levels of fibronectin, type IV collagen, and α-SMA were all up-regulated in renal cortex of the 5/6 Nx group. Administration of perindopril or JPYSF significantly reduced these fibrosis-associated proteins expression (Fig. 4a-d). Immunofluorescence (IF) analysis further proved above results (Fig. 4e).

### JPYSF up-regulated subunits of mitochondrial respiratory complex in 5/6 Nx rats

**Fig. 5:**
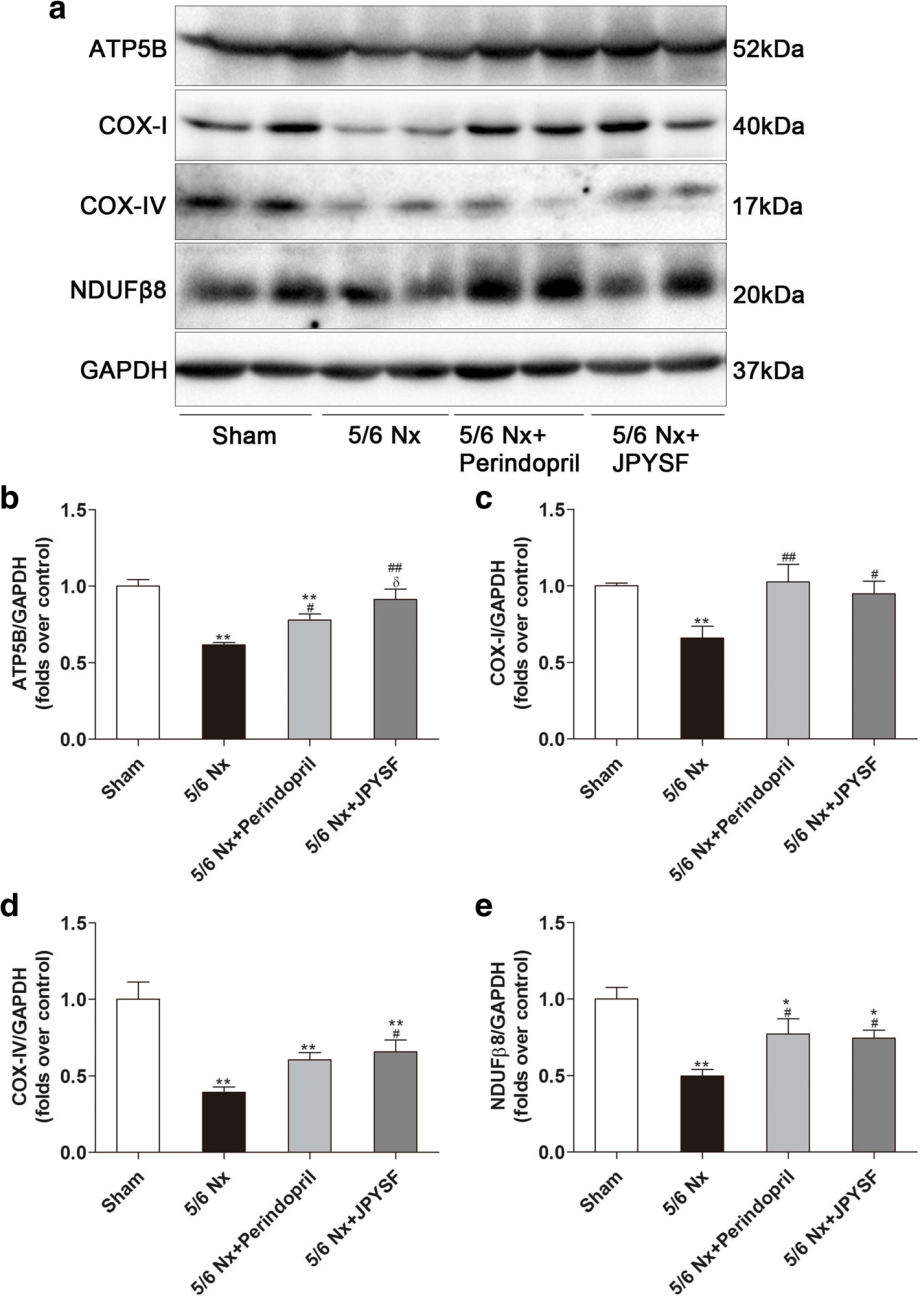
JPYSF improves mitochondrial function in 5/6 Nx rats. **a** Representative Western blot images indicate that ATP5B, COX-I, COX-IV, and NDUFβ8 protein expression were significantly reduced in the 5/6 Nx group but were increased by JPYSF treatment. **b**-**e** Densitometric analysis of ATP5B, COX-I, COX-IV, and NDUFβ8 protein expression normalized to GAPDH content. Data are presented as the means ± SEM, *n* = 6 rats per group. (*P < 0.05, **P < 0.01 compared with the sham group; ^#^*P* < 0.05, ^##^*P* < 0.01 compared with the 5/6 Nx group; ^δ^*P* < 0.05 compared with the 5/6 Nx + Perindopril group)

Producing adenosine triphosphate (ATP) via respiratory complex is an important function of mitochondrion. As shown in Fig. 5, protein levels of ATP5B, a subunit of mitochondrial ATP synthase, COX-I, COX-IV, and NDUFβ8, three subunits of mitochondrial respiratory complex, were all reduced in renal cortex of the 5/6 Nx group. These proteins expression were all restored in the 5/6 Nx + perindopril group and the 5/6 Nx + JPYSF group.

### JPYSF modulated mitochondrial quality control network in 5/6 Nx rats

**Fig. 6:**
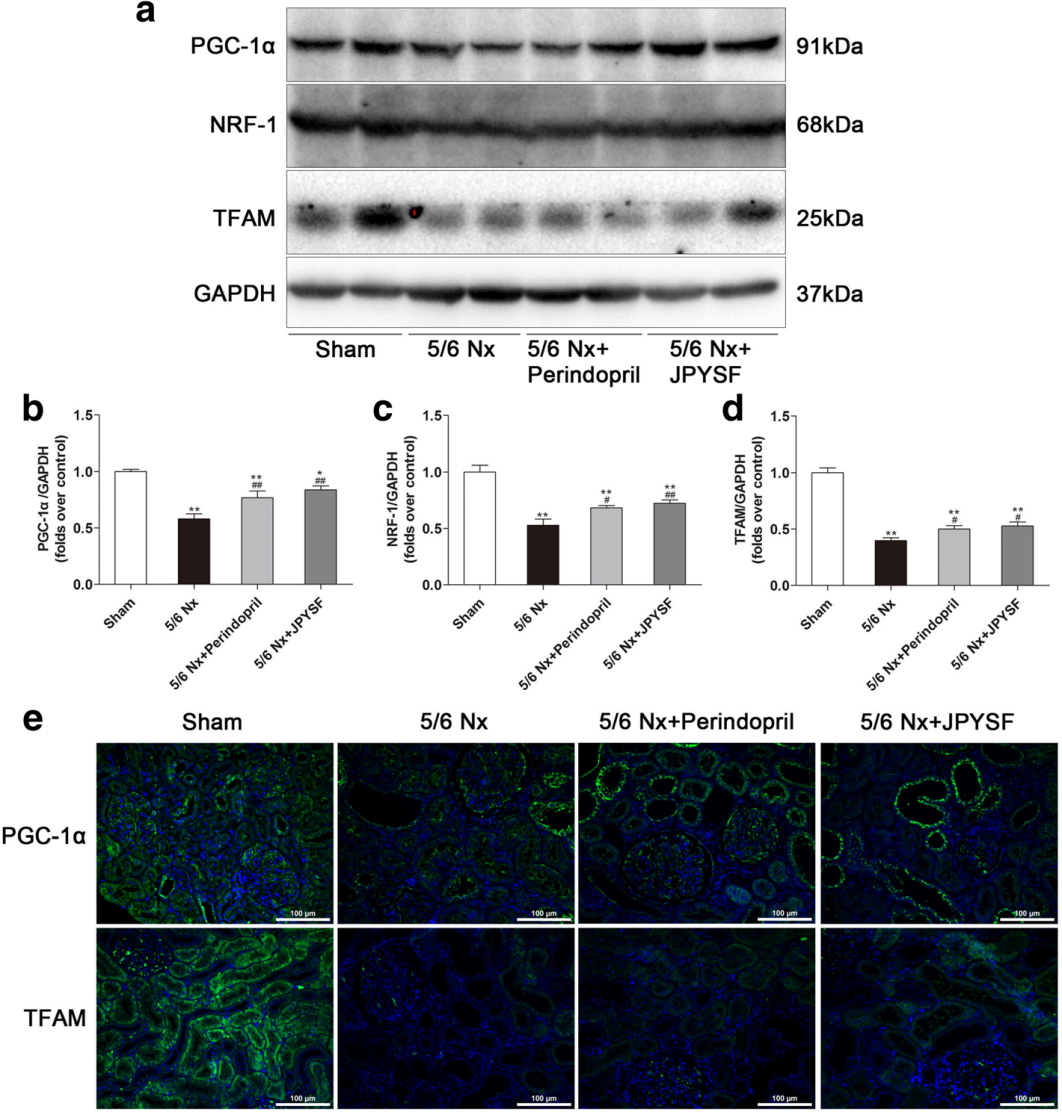
JPYSF increases mitochondrial biogenesis in 5/6 Nx rats. **a** Representative Western blot images show decreased PGC-1α, NRF-1, and TFAM expression in the 5/6 Nx group, which were increased by JPYSF treatment. **b**-**d** Densitometric analysis of PGC-1α, NRF-1, and TFAM protein expression normalized to GAPDH content. **e** Representative immunofluorescence images of PGC-1α and TFAM. Green corresponds to interest proteins, and blue corresponds to nuclear staining. All images are shown at identical magnification, × 200, scale bar = 100 μm. Data are presented as the means ± SEM, *n* = 6 rats per group. (*P < 0.05, **P < 0.01 compared with the sham group; ^#^*P* < 0.05, ^##^*P* < 0.01 compared with the 5/6 Nx group)

As shown in Western blot images (Fig. 6a-d), the protein abundance of PGC-1α, the master regulator of mitochondrial biogenesis, and its downstream signal NRF-1 and TFAM were significantly decreased in the 5/6 Nx group, whereas these protein levels were significantly increased following treatment of perindopril or JPYSF. The expression of PGC-1α and TFAM were further proved by IF analysis (Fig. 6e).

**Fig. 7:**
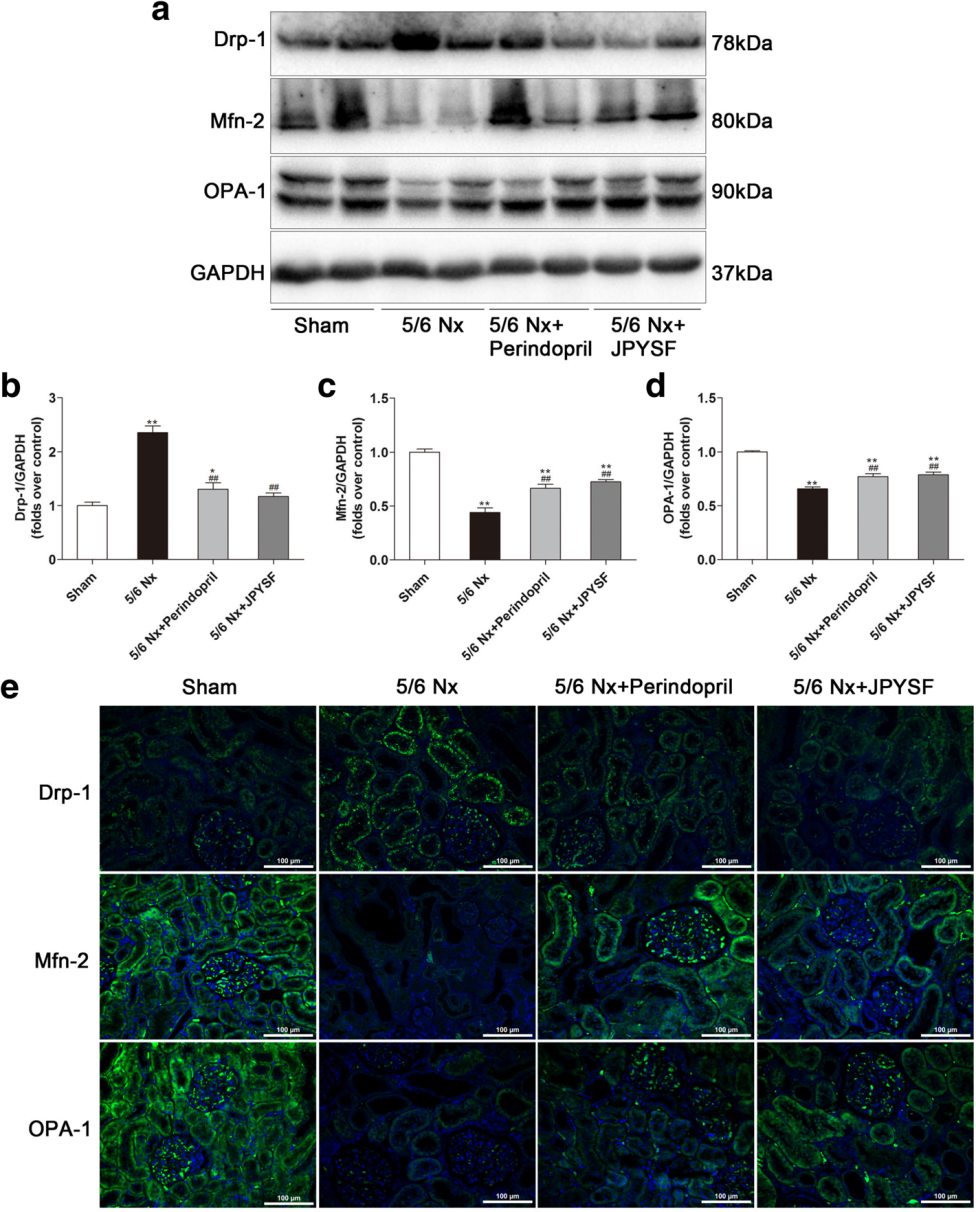
JPYSF decreases mitochondrial fission and increases mitochondrial fusion. **a** Representative Western blot images show increased Drp-1, decreased Mfn-2 and OPA-1 expression in the 5/6 Nx group, which were restored by JPYSF treatment. **b**-**d** Densitometric analysis of Drp-1, Mfn-2, and OPA-1 protein expression normalized to GAPDH content. **e** Representative immunofluorescence images of Drp-1, Mfn-2, and OPA-1. Green corresponds to interest proteins, and blue corresponds to nuclear staining. All images are shown at identical magnification, × 200, scale bar = 100 μm. Data are presented as the means ± SEM, *n* = 6 rats per group. (*P < 0.05, **P < 0.01 compared with the sham group; ^##^*P* < 0.01 compared with the 5/6 Nx group)

Mitochondrial morphology is governed by the balance between fission and fusion. Rats in the 5/6 Nx group presented increased mitochondrial fission and decreased mitochondrial fusion, as evidenced by up-regulation of Drp-1 and down-regulation of Mfn-2 and OPA-1 in Western blot analysis (Fig. 7a-d) and IF analysis (Fig. 7e).

**Fig. 8:**
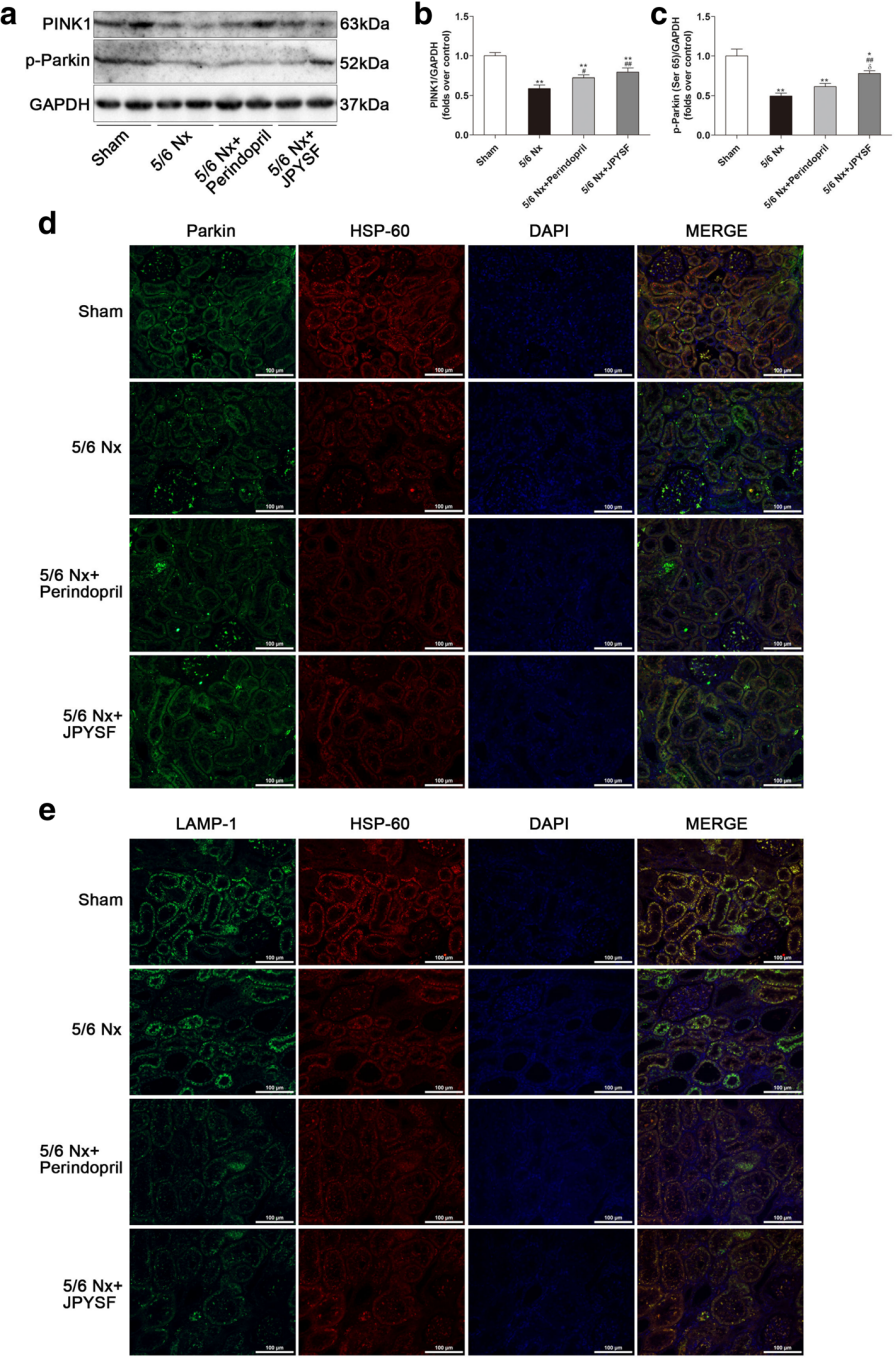
JPYSF restores the level of mitophagy in 5/6 Nx rats. **a** Representative Western blot images show decreased PINK1 and p-Parkin expression in the 5/6 Nx group, which were restored by JPYSF treatment. **b**, **c** Densitometric analysis of PINK1 and p-Parkin protein expression normalized to GAPDH content. **d** Representative immunofluorescence images indicating the colocalization of Parkin and HSP-60. **e** Representative immunofluorescence images indicating the colocalization of LAMP-1 and HSP-60. Green corresponds to interest proteins, red corresponds to mitochondria, and blue corresponds to nuclear staining. All images are shown at identical magnification, × 200, scale bar = 100 μm. Data are presented as the means ± SEM, *n* = 6 rats per group. (*P < 0.05, **P < 0.01 compared with the sham group; ^#^*P* < 0.05, ^##^*P* < 0.01 compared with the 5/6 Nx group; ^δ^*P* < 0.05 compared with the 5/6 Nx + Perindopril group)

PINK1/Parkin-mediated mitophagy is the best known mitophagy pathway in mammalian cell. PINK1 expression was overtly down-regulated in renal cortex of the 5/6 Nx group (Fig. 8a, b). PINK1 stabilization and accumulation in mitochondria would further recruit Parkin from the cytoplasm to the mitochondria and phosphorylate it at Serine 65. Western blotting showed that p-Parkin (Ser 65) was also down-regulated in renal cortex of the 5/6 Nx group (Fig. 8a, c). IF analysis indicated that less Parkin colocalized with HSP-60, a mitochondrial marker, in the 5/6 Nx group (Fig. 8d). Furthermore, the colocalization of HSP-60 and LAMP-1, a marker of lysosome, was also less in the 5/6 Nx group, compared with the sham group (Fig. 8e). Above data suggested that PINK1/Parkin-mediated mitophagy was down-regulated in the kidney of 5/6 Nx rats. Treatment of perindopril or JPYSF significantly restored PINK1/Parkin-mediated mitophagy in 5/6 Nx rats. Taken together, these data suggest that mitochondrial quality control network was disturbed in the kidney of 5/6 Nx rats, and could be restored by JPYSF treatment.

## Discussion

Accumulated evidence have been indicated that pathological mechanisms are associated with the excessive accumulation of extracellular matrix and podocyte loss and inflammation as well as abnormal lipid metabolism and amino metabolism in CKD patient and animal models [30–34]. However, the status of mitochondrial quality control network in CKD was less investigated. In the present study, we successfully reproduced characteristics of CKD in a rat model of 5/6 Nx, as evidenced by decreased kidney function, proteinuria, and damaged kidney structure. A traditional Chinese herbal formula JPYSF could improve kidney function; ameliorate proteinuria and renal pathological injury. Furthermore, we found obvious mitochondrial dysfunction in 5/6 Nx rats accompanied by disturbed mitochondrial quality control network, which could be restored and modulated by treatment of JPYSF. In the present study, JPYSF and perindopril have comparable effects on improving kidney function. However, our previous studies have demonstrated that JPYSF could induce erythropoietin expression [35], which is related with renal anemia, and improve muscle atrophy in 5/6 Nx rats [36]. Thus, the effect of JPYSF seems to be holistic and multi-target, which are advantages of JPYSF compared with perindopril.

Renal fibrosis is the final common pathway by which earlier stages of CKD progress to end-stage renal disease. Anti-fibrotic therapy is an attractive approach to treat patients with CKD. In our study, administration of JPYSF protected kidneys from fibrotic injury by significantly down-regulating expression of fibronectin, type IV collagen, and α-SMA in 5/6 Nx rats. Astragali Radix, the ‘sovereign medicinal’ in JPYSF, has been previously found to have anti-fibrotic effects through inhibition of the transforming growth factor-β1 pathway in several cell types and tissues [37–39]. Astragaloside IV, the active ingredient isolated from Astragali Radix and confirmed in JPYSF extract by HPLC-MS analysis (Fig. 1), also has been reported to ameliorate renal fibrosis in vivo and in vitro [40, 41]. In addition, Salviae Miltiorrhizae Radix et Rhizoma and Rhei Radix et Rhizoma, the ‘courier medicinal’ of JPYSF, have been observed to decrease levels of kidney extracellular matrix in diabetic *db/db* mice [42], and prevent renal fibrosis by inhibiting epithelial-mesenchymal transition in HgCl_2_-induced rat model [43]. Apart from three herbs mentioned above, the other components of JPYSF were less reported to have reno-protective effect. However, it is hard to tell which herb most likely conferred the observed therapeutic effects on our CKD model. This may be an orchestrated effect. TGF-β/Smad signaling plays pivotal role in the development and progression of renal fibrosis. Previous studies have reported that the main components of JPYSF, including Huang-Qi [44], Dan-Shen [45], and Da-Huang [42], could modulate TGF-β/Smad signaling pathway in renal or liver fibrosis. It is speculated that TGF-β/Smad signaling may be involved in the anti-fibrosis effect of JPYSF.

Mitochondria are primarily responsible for producing ATP via oxidative phosphorylation in the inner mitochondrial membrane. However, mitochondria can rapidly change into death-promoting organelles by producing excessive reactive oxygen species and releasing prodeath proteins, which will result in disrupted ATP synthesis and activation of cell death pathways [46]. These characteristics of mitochondrion place it in the central position of pathogenesis of metabolic disease, neurodegenerative disease, and cancer [47]. Kidneys are fuel-hungry organs and only second to the heart in mitochondrial number and oxygen consumption [48]. Therefore, mitochondrial dysfunction in the kidneys plays a critical role in the pathogenesis of multiple kidney diseases [18]. In our study, mitochondrial respiratory complex subunits and ATP synthase subunit were all downregulated in 5/6 Nx rats, which indicated disturbed mitochondrial function in CKD. Similar with our results, another study found that COX-IV level was significantly reduced in rat kidneys after 5/6 Nx insult [49]. More significantly, we found obvious derangement of mitochondrial quality control network in 5/6 Nx rats, presenting as decreased mitochondrial biogenesis, increased mitochondrial fission, decreased mitochondrial fusion, and decreased mitophagy. Mitochondrial quality control network is responsible for mitochondrial homeostasis and mitochondrial function [27]. Our results showed that JPYSF could restore the disturbed protein expression associated with mitochondrial quality control network in 5/6 Nx rats, which may be contribute to its effect in improving mitochondrial function. Wallace suggests that mitochondria may be considered as Qi (Chi) [50], which loosely translates as vital force or energy, according to its TCM interpretation. Astragali Radix, the ‘sovereign medicinal’ in JPYSF, is one of the most important drugs for ‘replenishing vital energy’ in TCM. And our unpublished data showed that astragaloside IV, the mainly active component of Astragali Radix, could reduce mitochondrial fission in diabetic *db/db* mice. Astragaloside IV has also been reported to increase PGC-1α expression in vascular smooth muscle cells [51] and rat heart [52]. Thus, it is possible that Astragali Radix in JPYSF most likely affected mitochondrial quality control. Previous studies demonstrated that astragaloside IV [53] and salvianolic acid B [54] could modulate mammalian target of rapamycin (mTOR) signaling pathway, which is close related with mitochondrial quality control. Further study is needed to explore the underlying mechanism of JPYSF in regulating mitochondrial quality control. Collectively, our study shed lights on the regulative effect of TCM formula on mitochondrial quality control network in CKD, which echoes a previous review demonstrated the role of TCM in cardiovascular disease by regulating the structure and function of mitochondria [55]. However, more rigorous pharmacologic studies and detailed mechanistic studies using modern scientific methodology and approaches are needed to elucidate the therapeutic potential of TCM for CKD.

## Conclusions

In conclusion, the present study demonstrates that orally administered JPYSF significantly retards development and progression of CKD in a 5/6 Nx rat model. Our results also suggest that modulating mitochondrial quality control network may be related with the beneficial effect of JPYSF on CKD.

## Abbreviations

5/6 Nx: 5/6 nephrectomy
ALT: alanine transaminase
AST: aspartate transaminase
ATP: adenosine triphosphate
ATP5B: ATP synthase subunit beta
BUN: blood urea nitrogen
CKD: chronic kidney disease
COX-I: cytochrome c oxidase subunit I
COX-IV: cytochrome c oxidase subunit IV
DAPI: 4′,6-diamidino-2-phenylindole
DHGCT: Da-Huang-Gan-Cao-Tang
Drp-1: dynamin-related protein 1
GAPDH: glyceraldehyde-3-phosphate dehydrogenase
HRP: horseradish peroxidase
HSP-60: heat shock protein-60
JPYSF: Jian-Pi-Yi-Shen Formula
LAMP-1: lysosomal-associated membrane protein 1
Mfn-2: mitofusin 2
NAG: N-acetyl-β-D-glucosaminidase
NDUFβ8: nicotinamide adenine dinucleotide dehydrogenase (ubiquinone)-1β subcomplex 8
NRF-1: nuclear respiratory factor 1
OPA-1: optic atrophy 1
PAS: periodic acid-Schiff
PGC-1α: peroxisome proliferator-activated receptor-γ coactivator-1α
PINK1: PTEN-induced putative kinase 1
TCM: traditional Chinese medicine
TFAM: mitochondrial transcription factor A
YPFS: Yu-Ping-Feng-San; Scr serum creatinine
α-SMA: α-smooth muscle actin
